# Hypoxia Modulates Sodium Chloride Co-transporter via CaMKII-β Pathway: An In Vitro Study with mDCT15 Cells

**DOI:** 10.3390/life14101229

**Published:** 2024-09-25

**Authors:** Lijuan Liang, Kohei Ueda, Sayoko Ogura, Tatsuo Shimosawa

**Affiliations:** 1 Department of Clinical Laboratory, International University of Health and Welfare, Chiba 286-8686, Japan; lianglijuan0306@163.com; 2 Department of Physiology, International University of Health and Welfare, Chiba 286-8686, Japan; kueda@iuhw.ac.jp; 3 Department of Pathology and Microbiology, Division of Laboratory Medicine, Nihon University School of Medicine, Tokyo 173-8610, Japan; ogura.sayoko@nihon-u.ac.jp

**Keywords:** hypoxia, cobalt chloride, sodium-chloride co-transporter, CaMKII-β

## Abstract

Background: Hypoxia plays a crucial role in regulating various cellular functions, including ion-transport mechanisms in the kidney. The sodium-chloride co-transporter (NCC) is essential for sodium reabsorption in the distal convoluted tubule (DCT). However, the effects of hypoxia on NCC expression and its regulatory pathways remain unclear. We aimed to explore the effects and potential mechanisms of hypoxia on NCC in vitro. Methods: mDCT15 cells were treated with cobalt chloride (CoCl_2_) at a concentration of 300 μmol/L to induce hypoxia. The cells were harvested at different time points, namely 30 min, 1 h, 6 h, and 24 h, and the expression of NCC and CaMKII-β was analyzed using Western blot. Results: A time-dependent upregulation of NCC and CaMKII-β expression in response to CoCl_2_-induced hypoxia. KN93 reversed the effect of CoCl_2_ on NCC and phosphorylated NCC expression. Conclusions: Hypoxia, mediated through cobalt chloride treatment, upregulates NCC expression via the CaMKII-β pathway in mDCT15 cells.

## 1. Introduction

The distal convoluted tubule (DCT) in the kidney is essential for maintaining electrolyte homeostasis by reabsorbing sodium and chloride ions [1]. The sodium-chloride co-transporter (NCC) is a crucial membrane protein involved in renal sodium reabsorption. The regulation of NCC activity is vital for maintaining blood-pressure homeostasis, and its dysregulation is associated with several pathophysiological conditions, including hypertension [2]. The regulation of NCC is widely investigated [3]. Oxidative stress, angiotensin II as well as potassium are potent regulators. On the other hand, the kidney is under hypoxic conditions and high in oxidative stress [4]; therefore, this physiological condition may affect NCC activity.

Hypoxia plays a significant role in various renal diseases, such as chronic kidney disease and acute kidney injury, which are often accompanied by reduced oxygen supply and increased oxidative stress [5]. Early studies have indicated that despite receiving a large portion of the cardiac output (approximately 25% in humans), certain areas of the kidney remain physiologically hypoxic [5]. Compared to other organs, the oxygen content in the kidney is relatively low, making it more responsive to further reductions in oxygen supply [6,7]. Sodium reabsorption is the primary determinant of renal oxygen consumption [8]. NCC, located in the distal convoluted tubule, is a key regulator of sodium reabsorption. Hypoxia may alter sodium handling in the kidney by affecting NCC expression and phosphorylation. Therefore, hypoxia is a crucial factor to consider when studying NCC expression and phosphorylation.

Recently, we reported that calcium/calmodulin-dependent protein kinase II (CaMKII) is another regulator of phosphorylation of NCC [9]. While this previous study suggested CaMKII’s role in the kidney, none have explicitly demonstrated its functional impact in the DCT. CaMKII is well studied in neural function and predominantly functional in non-epithelial cells [10], but its role in epithelial cells, such as those in the DCT, remains less explored. CaMKII was first discovered in the brain and subsequently shown to play a critical role in synaptic plasticity in the central nervous system [11]. It exists in four isoforms (α, β, γ, δ), with the α and β isoforms being predominant in the nervous system [12]. It plays a critical role in various physiological functions, including cell-cycle regulation, apoptosis, gene expression, neurotransmission, synaptic plasticity, long-term potentiation, memory formation, and responses like early depolarization and aortic contraction [13]. These functions highlight CaMKII’s importance in both neural and vascular systems. But, in our study, CaMKII is physiologically important in epithelial cells and regulates NCC activation.

In this study, we aim to investigate whether hypoxic conditions affect NCC phosphorylation via CaMKII in mouse DCT15 (mDCT15) cells, which is an established model of the distal convoluted tubule [14]. We applied a cobalt chloride (CoCl_2_) model that mimics hypoxic conditions, and an increase in HIF-1α levels was observed [15]. HIF-1α is rapidly degraded under normoxic conditions. Two proline residues in its oxygen-dependent degradation domain are hydroxylated by prolyl hydroxylases (PHD), leading to ubiquitination mediated by the von Hippel-Lindau tumor-suppressor protein (pVHL), which targets HIF-1α for proteasomal degradation [16]. Under hypoxic conditions, the prolyl hydroxylation process is inhibited, resulting in the stabilization and accumulation of HIF-1α, which in turn triggers the hypoxic response [17]. HIF-1α helps cells adapt to hypoxia by regulating various genes involved in cell metabolism, angiogenesis, and survival [18]. In kidney diseases, HIF-1α protects renal tissue from hypoxic damage by promoting angiogenesis, regulating metabolism, and reducing inflammation [19].

We confirmed that cobalt chloride treatment successfully induced hypoxia-related cellular responses by detecting the expression of HIF-1α, laying the foundation for subsequent studies on the changes in NCC expression.

Based on this result, we further examined the temporal changes in NCC and CaMKII-β expression following CoCl_2_ treatment at different time points (30 min, 1 h, 6 h, and 24 h). Furthermore, phosphorylated NCC (p-NCC) is the active form of NCC, and phosphorylated CaMKII-β (p-CaMKII) represents the activated form of CaMKII-β. Therefore, we also examined the levels of p-NCC and p-CaMKII-β in our study. The phosphorylation of NCC plays a critical role in its regulation. Phosphorylation activates NCC, facilitating its movement to the plasma membrane, where it can participate in sodium reabsorption. This process is crucial for maintaining electrolyte balance and blood-pressure regulation.

Additionally, we explore the role of CaMKII in NCC regulation using KN93(N-[2-[N-(4-chlorocinnamyl)-N-methylaminomethyl]phenyl]-N-(2-hydroxyethyl)-4-methoxybenzenesulfonamide) to inhibit CaMKII activity. KN93 is the most widely used inhibitor for studying the cellular and in vivo functions of CaMKII [20]. It binds directly to Ca^2+^/CaM and not to CaMKII. This binding would disrupt the ability of Ca^2+^/CaM to interact with CaMKII, effectively inhibiting CaMKII activation [21]. By inhibiting CaMKII activity, we were able to assess its specific role in NCC regulation under hypoxic conditions.

## 2. Materials and Methods

### 2.1. Experimental Protocol

To investigate the effect of hypoxia on sodium-chloride co-transporter (NCC) in vitro, we treated mDCT15 cells with cobalt (II) chloride (CoCl_2_) (300 μmol/L) (Cat.035-10982, FUJIFILM, Tokyo, Japan) [22]. The cells were collected after 30 min,1 h, 6 h, and 24 h to investigate the expression of NCC and CaMKII-β by Western blot.

### 2.2. Cell Culture

mDCT15 cells were provided by Dr Robert S Hoover [14] were cultured on 10 cm dish (Cat.FG-2090, Genetics, Tokyo, Japan) in a low glucose Dulbecco’s Modified Eagle Medium (Cat.08456-36, NACALAI TESQUE, Kyoto, Japan) supplemented with 5% heat-inactivated fetal bovine serum (REF.35-010-CV, Corning, NY, USA) and 1% penicillin-streptomycin (Cat.26253-84, NACALAI TESQUE, Kyoto, Japan) at 37 °C. The cells were used for experiments when they reached 80% confluence (To prevent cell death due to excessive fusion, cells cultured with CoCl_2_ for 24 h were incubated at 40% confluence).

### 2.3. Drug Treatments

KN93(CAS:1188890-41-6, FUJIFILM, Tokyo, Japan, 10 mM stock in DMSO; dimethyl sulfoxide) in a dose of 10 μmol/L was used to block CaMKII [23]. For co-treatment, cells were first incubated with KN93 for 30 min, followed by incubation with CoCl_2_.

### 2.4. SDS-PAGE and Western Blot Analysis

The plasma membrane protein (2 × 10^7^ cells) was extracted with a Minute Plasma membrane protein extraction kit for animal cultured cells and tissues (Cat.SD-001/SN-002, Invent Biotechnologies, Plymouth, MN, USA) without boiling for NCC expression. The protein concentration was measured using a Pierce™ BCA Protein Assay Kit (Cat.23225, Thermo Fisher Scientific, Rockford, IL, USA). After electrophoresis, TGX^TM^ gels (Cat.456-9036, Bio Rad, CA, USA) were transferred to polyvinylidene difluoride membranes on ice. After membranes were blocked with PVDF Blocking Reagent Set (Cat. NYPBR, TOYOBO, Osaka, Japan) for 60 min, they were probed with various primary antibodies overnight at 4 °C. This was followed by incubation with secondary antibodies (anti-Mouse-HRP, anti-Sheep-HRP, or anti-Rabbit-HRP) in Can Get Signal reagent (Cat. No. NKB101, TOYOBO, Osaka, Japan) for 1 h at room temperature and subsequently visualized by enhanced chemiluminescence ImmunoStar LD (Cat.290-69904, Fujifilm, Tokyo, Japan). The signals on immunoblots were detected with the ChemiDoc MP Imaging System. Antibodies for detection of NCC (AB3553, Millipore, Darmstadt, Germany), NCC Phospho Thr 46 + Thr 50 + Thr 55 (Sheep No. S908B, University of Dundee, MRC Protein Phosphorylation and Ubiquitylation Unit), CAMKII-β (Cat.ab34703, Abcam, Cambridge, UK), and Phospho-CaMKII alpha/beta/delta (Thr305) (Cat. PA5-37832, Thermo Fisher Scientific, Rockford, IL, USA). For normalization, the membranes were stripped and re-probed with either β-actin (Cat.B0556-2 mL, Sigma Aldrich, St. Louis, MO, USA) or Na-K ATPase (Cat. 05-369, Sigma Aldrich, St. Louis, MO, USA).

### 2.5. Statistical Analysis

Statistical analyses were performed using SPSS software (IBM SPSS Statistics 29.0.2.0, Armonk, NY, USA), and all data are expressed as mean ± SD. When appropriate, comparisons among groups were analyzed using one-way analysis of variance or ANOVA for repeated measurement followed by Dunnett’s or Tukey-Kramer test. *p* < 0.05 was considered significant. Western blot band was calculated for its density by Image J software (ImageJ 1.52i, Bethesda, MD, USA) and at least five data sets were used for progressive comparisons in scatter plots.

## 3. Results

### 3.1. CoCl_2_ Incubation Induced Hypoxia-Inducible Factor-1α (HIF-1α) in mDCT15 Cells

**Figure 1:**
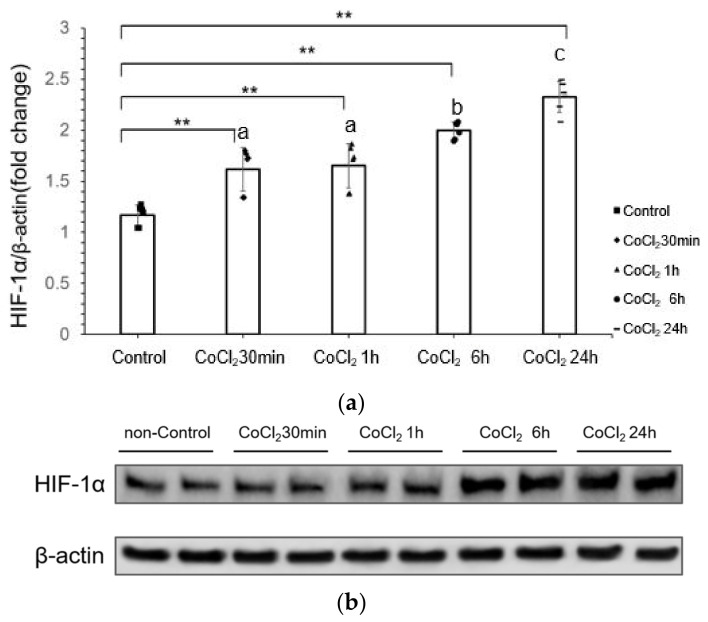
HIF-1α expression levels at different CoCl_2_ incubation times (30 min, 1 h, 6 h, and 24 h). (**a**) Quantitative analysis of HIF-1α expression at different CoCl_2_ incubation time points; (**b**) Representative Western blot images showing HIF-1α expression at different CoCl_2_ incubation times. All protein levels were normalized to β-actin. Cells were treated with 300 µM CoCl_2_. Data are presented as the mean ± SD. Error bars represent the standard deviation (*n* = 3 biological replicates, *t* = 6 technical replicates). Western blot band was calculated for its density by Image J software. Statistical analysis was performed using repeated measures ANOVA, followed by Dunnett’s post-hoc test for comparisons with the control group. * *p* < 0.05, ** *p* < 0.01, compared to the control. Pairwise comparisons between treatment groups were also performed using Tukey’s post-hoc test. Different letters indicate significant differences between groups (*p* < 0.05). The 30-min group and the 1-h group showed no significant difference, thus both are labeled with the same letter, a. The 6-h group is labeled b, and the 24-h group is labeled c, indicating significant differences from other groups.

Cobalt chloride is a common chemical reagent that can mimic a hypoxia environment [24]. We first investigated the HIF-1α expression with CoCl_2_ incubation to confirm if CoCl_2_ incubation induced hypoxia-related signaling. The results showed that the difference between each treatment group and the control group was significant (*p* < 0.001) (Figure 1a,b). The expression of HIF-1α increased gradually in a time-dependent manner. Between the treatment group CoCl_2_ 30 min to 1 h group difference was not significant (*p* = 0.995), but with 6 h and 24 h group difference was significant (*p* < 0.001). The difference between the CoCl_2_ 1- and 6-h and 24-h group was significant (*p* = 0.009 and *p* < 0.001). In general, with the increase of CoCl_2_ incubation time, the expression of HIF-1α significantly increased, especially in 6 h and 24 h CoCl_2_ treatment.

### 3.2. CoCl_2_ Incubation Induced NCC Activity and CAMKII-β Activity in mDCT15 Cells

**Figure 2:**
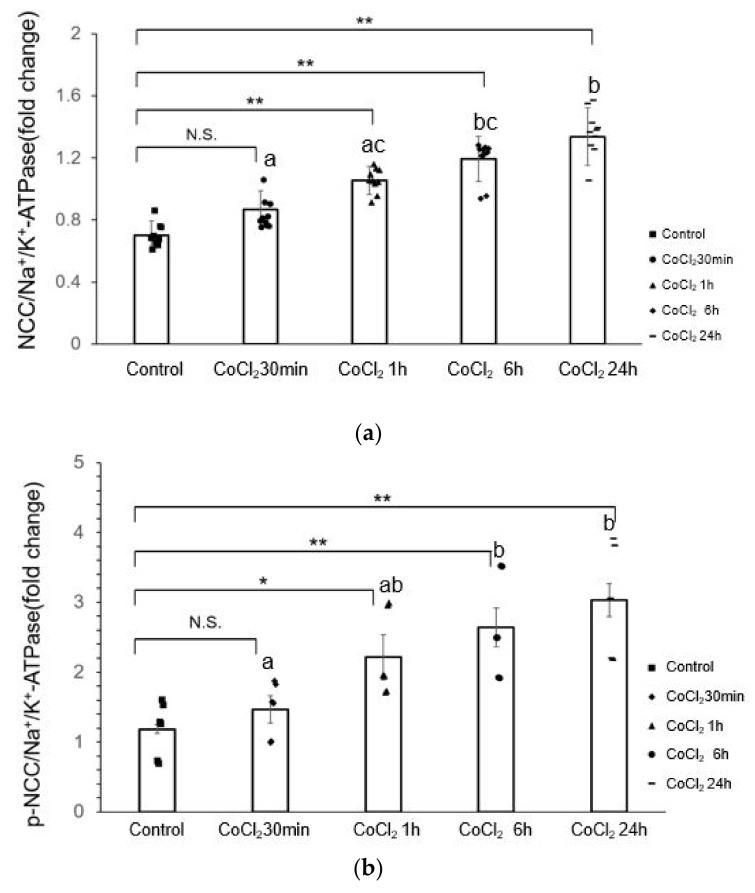

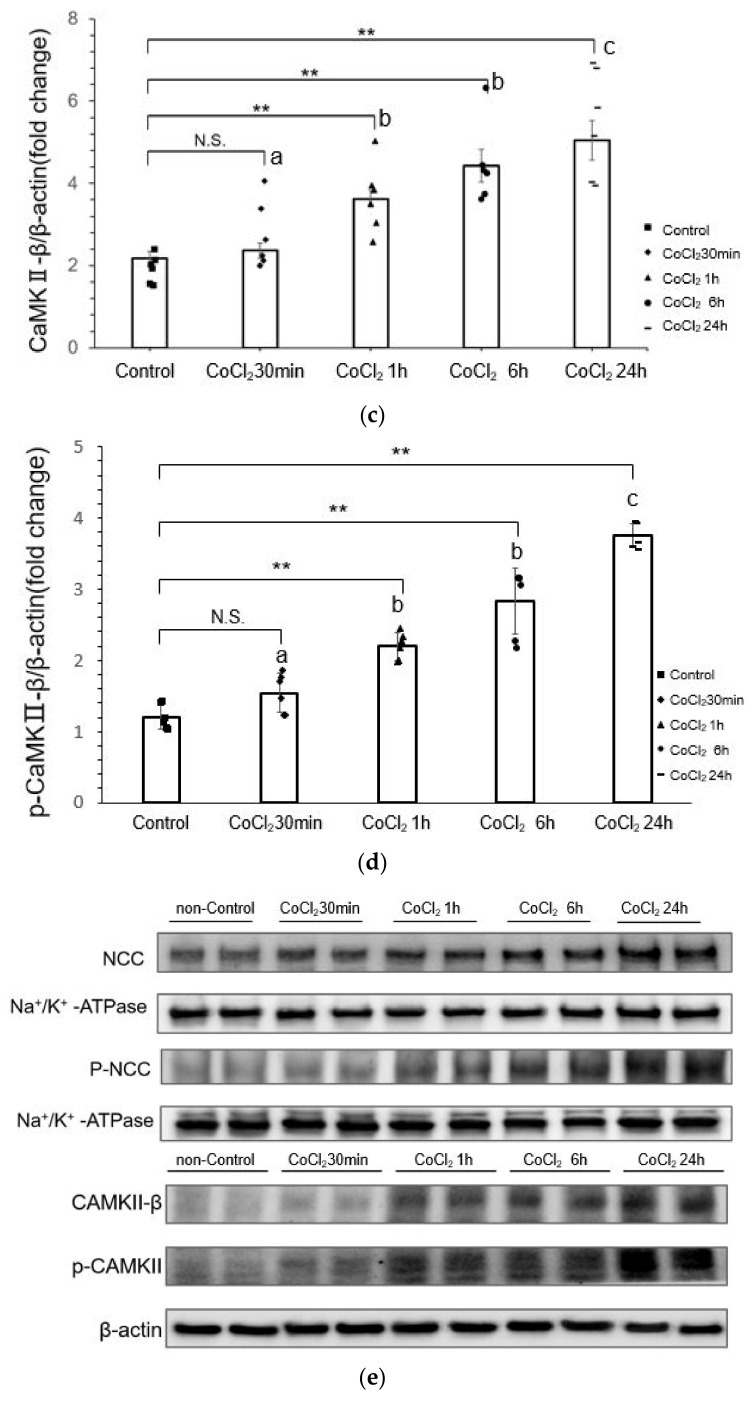
CoCl_2_ incubation induces changes in NCC expression, CAMKII-β expression, and phosphorylation in mDCT15 cells. (**a**) NCC expression significantly increased after 1 h of CoCl_2_ treatment, showing a time-dependent effect. (**b**) p-NCC expression significantly increased after 1 h of CoCl_2_ treatment; (**c**) CAMKII-β expression increased significantly after 1 h of CoCl_2_ treatment; (**d**) p-CAMKII expression significantly increased after 1 h of CoCl_2_ treatment. (**e**) Representative Western blot images showing NCC, p-NCC, CAMKII-β, and p-CAMKII expression in mDCT15 cells following CoCl_2_ treatment. All protein levels were normalized to Na^+^/K^+^-ATPase (for NCC and p-NCC) and β-actin (for CAMKII-β and p-CAMKII). Cells were treated with 300 µM CoCl_2_ for 30 min, 1 h, 6 h, and 24 h. Data are presented as the mean ± SD. Error bars represent the standard deviation (n = 3–5 biological replicates, t = 6–10 technical replicates). Western blot band was calculated for its density by Image J software. Statistical analysis was performed using repeated measures ANOVA, followed by Dunnett’s post-hoc test for comparisons with the control group. * *p* < 0.05, ** *p* < 0.01, compared to the control, N.S, not significant. Pairwise comparisons between treatment groups were also performed using Tukey’s post-hoc test. Different letters indicate significant differences between groups (*p* < 0.05).

We used CoCl_2_ to stimulate the mDCT15 cells to check the NCC activation by measuring membrane-bound NCC and CaMKII-β expression. As shown in Figure 2, CoCl_2_ incubation increased membrane-bound NCC and CaMKII-β expression.

There was no significant difference in membrane-bound NCC after 30 min of CoCl_2_ treatment compared with the control group (*p* = 0.316), indicating that short periods of CoCl_2_ treatment did not have a significant effect on NCC activation. Of note, the membrane-bound NCC was significantly increased after 1 h of CoCl_2_ treatment (*p* = 0.020) and after 24 h of CoCl_2_ treatment (*p* = 0.004) in line with the above-mentioned HIF1alpha expression, indicating that long-term CoCl_2_ treatment had a highly significant promoting effect on NCC activation (Figure 2a,e).

We also checked the phosphorylated NCC (p-NCC), an activated form of NCC, directly using a specific antibody and showed that with the extension of CoCl_2_ treatment time, the expression of p-NCC gradually increased (Figure 2b,e). p-NCC expression was significantly increased after 1 h of CoCl_2_ treatment (*p* = 0.008). The differences between 1 h and 6 h (*p* = 0.715) and 24 h (*p* = 0.148) were not significant, indicating that p-NCC expression was stable after 1 h of CoCl_2_ treatment.

The expression of CAMKII-β increased in a time-dependent fashion by CoCl_2_ treatment (Figure 2c,e). All treatment times (1 h, 6 h, and 24 h) showed a significant increase in CAMKII-β expression compared to the control group, except for the 30-min treatment group (*p* = 0.68).

At the same time, significant increases were observed in the level of p-CAMKII in CoCl_2_ treatment groups (1 h, 6 h, and 24 h) compared to the control group. This suggests a time-dependent effect of CoCl_2_ on p-CAMKII expression, with more extended treatment times resulting in a more significant increase in expression (Figure 2d,e). However, the 30-min treatment did not show a significant difference (*p* = 0.262) compared to the control group, indicating that extended exposure to CoCl_2_ is necessary for a significant increase in the level of p-CAMKII.

### 3.3. KN93 Inhibits the CoCl_2_-Induced Activity of NCC In Vitro via the CaMKII Pathway

To investigate the possible CaMKII pathway in CoCl_2_-induced hypoxia, we used KN93, a CaMKII inhibitor, and evaluated its impact on the NCC activation by membrane-bound and phosphorylated NCC.

**Figure 3:**
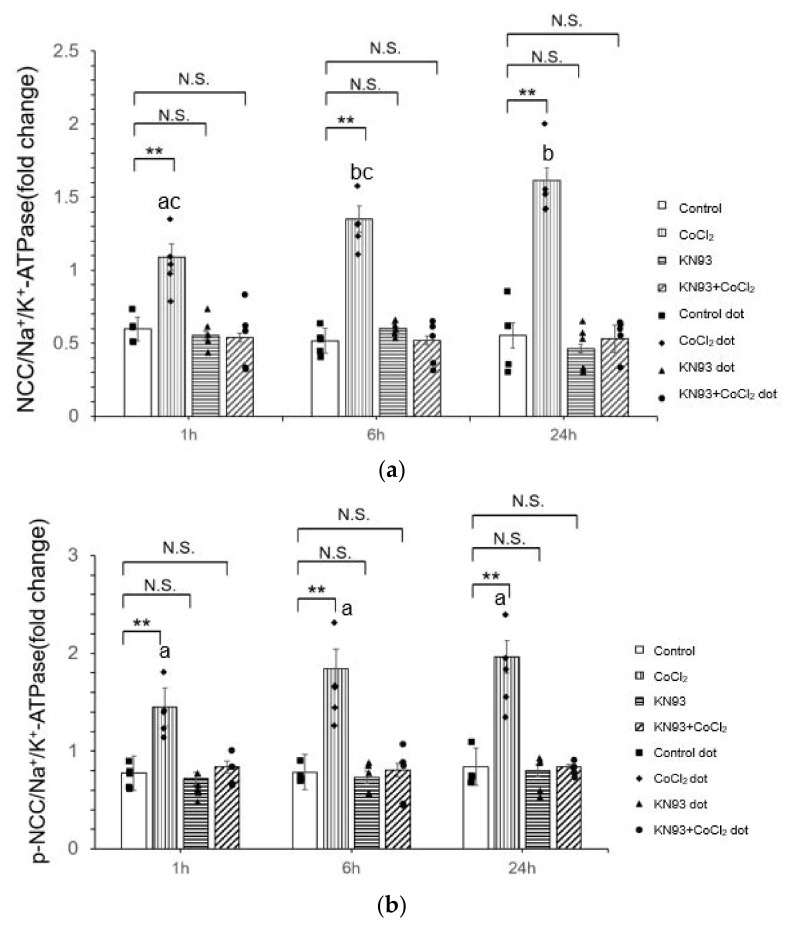

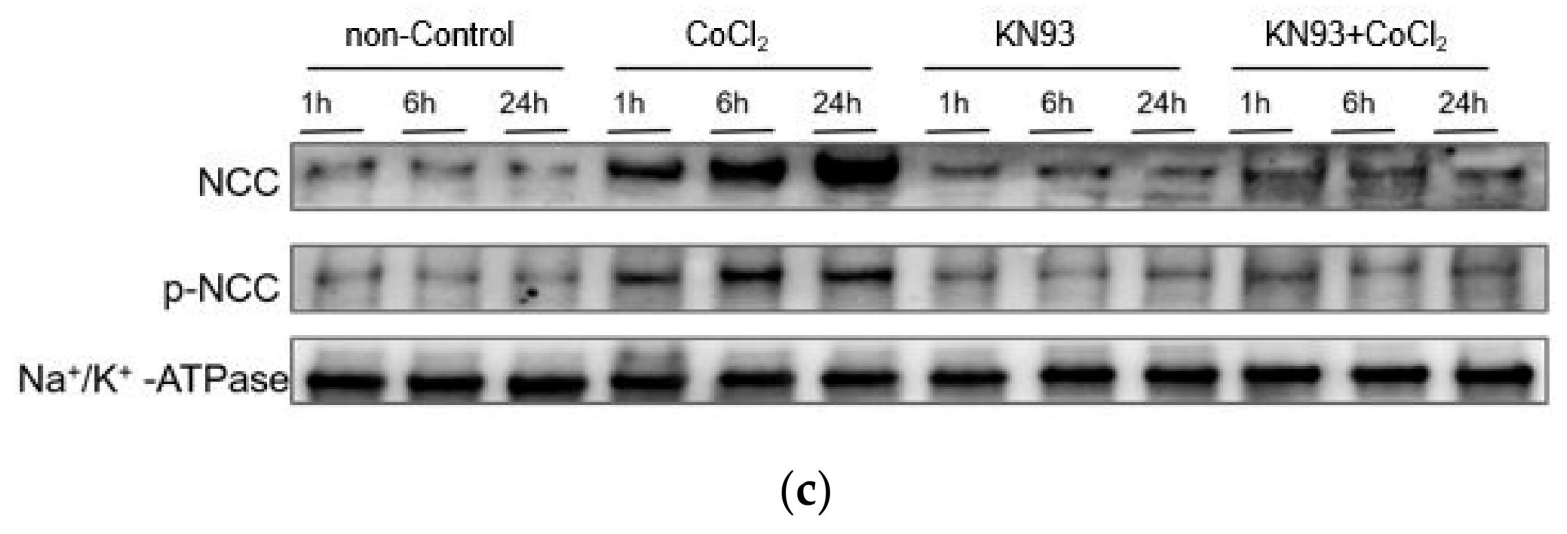
Effects of KN93 on CoCl_2_-induced NCC and p-NCC expression in mDCT15 cells. (**a**) NCC expression had no significant change with KN93 treatment and KN93+CoCl_2_ treatment; (**b**) p-NCC expression had no significant with KN93 treatment and KN93+CoCl_2_ treatment. (**c**) Representative Western blot images showing NCC and p-NCC expression in mDCT15 cells following KN93 and CoCl_2_ treatment. All protein levels were normalized to Na+/K+-ATPase. Cells were treated with 300 µM CoCl_2_ and 10 µM KN93 for 1 h, 6 h, and 24 h. For co-treatment, cells were first incubated with KN93 for 30 min, followed by incubation with CoCl_2_. Data are presented as the mean ± SD. Error bars represent the standard deviation (n = 5 biological replicates, t = 10 technical replicates). Western blot band was calculated for its density by Image J software. Statistical analysis was performed using repeated measures ANOVA, followed by Dunnett’s post-hoc test for comparisons with the control group. * *p* < 0.05, ** *p* < 0.01, compared to the control, N.S, not significant. Pairwise comparisons between treatment groups were also performed using Tukey’s post-hoc test. Different letters indicate significant differences between groups (*p* < 0.05).

To ensure CaMKII was fully suppressed in the KN93 and CoCl_2_ co-treatment group, we incubated KN93 for 30 min first and then incubated with CoCl_2_. In groups without treatment by CoCl_2_, KN93 did not result in significant changes in the membrane-bound expression of NCC (Figure 3a,c) and p-NCC (*p* > 0.05) (Figure 3b,c). KN93 reversed the effect of CoCl_2_ on NCC and p-NCC expression. This suggests that CaMKII is the upstream of NCC activation by hypoxia.

## 4. Discussion

Recently, we studied lcn2 knockout (lcn2 KO) mice fed a low-sodium diet (0Na) and found that acute administration of rlcn2 increased the phospho-T287/total CaMK2β and phospho-T53/total NCC protein ratio in lcn2 KO mice, but not in wild-type (WT) mice [9]. This study focused on the effects of lcn2 and sodium intake on NCC phosphorylation in a salt-sensitive hypertension model, while our study extends these findings to a different context: CoCl_2_-induced hypoxia, which induces NCC phosphorylation in mDCT15 cells via the CaMKII pathway. By focusing on hypoxia rather than sodium consumption or lcn2, our study provides new insights into how environmental stressors such as hypoxia regulate NCC through CaMKII activation.

In this study, we mimic a hypoxic condition using CoCl_2_ in a mouse distal convoluted cell line (mDCT15 cell) and showed that hypoxia activates CaMKII signaling in the kidney to activate NCC by its phosphorylation. The results demonstrated that CoCl_2_ treatment significantly upregulated the membrane-bound NCC and its phosphorylated form, p-NCC, and increased the expression of CaMKII-β and its phosphorylated form, p-CaMKII. Additionally, the inhibition of CaMKII with KN93 reversed NCC phosphorylation, suggesting a critical role of CaMKII in NCC regulation under hypoxic conditions. These findings suggest that the CoCl_2_-induced hypoxic environment may regulate the expression of NCC through the activation of the CaMKII signaling pathway.

It has been reported that hypoxia regulates several ion channels in the kidney and NCC is also one of them [25,26]. As an essential transporter in the renal distal tubule, the increased expression of NCC may help to maintain intracellular sodium balance and play a key role in the adaptation mechanism of cells to hypoxic conditions. However, the signaling mechanisms are not fully known.

Using KN93 revealed that NCC activation under CoCl_2_ treatment was reversed in parallel with the reduction of CaMKII. This indicates that CaMKII is in the upstream of the regulation of NCC under hypoxic conditions. These findings are the first report that the role of CaMKII is a key molecular regulator of NCC expression in low oxygen environments.

We speculate that under hypoxic conditions, the induction of NCC expression by CoCl_2_ is mediated through the activation of CaMKII signaling pathways. In non-epithelial cells, it has been reported that CaMKII is closely related to hypoxic stress, not only in neuronal cells [27] but cardiac and vascular smooth muscle cells [28,29,30]. Kratimenos et al. [27] have used experimental and computational approaches to determine the significance of the SrcCa^2+^/CaM pathway in neuronal excitotoxicity in a large animal model of neonatal hypoxic brain injury. In vitro hypoxia and reoxygenation models of myocardial cells, it has been reported that Suberoylanilide hydroxamic acid (SAHA) treatment alleviated myocardial cell apoptosis as well as mitochondrial dysfunction resulting from myocardial ischemia-reperfusion (I/R) impairment, and contributed to myocardial function recovery by inhibiting the Na⁺-Ca^2^⁺ exchanger (NCX)-Ca^2+^-CaMKII pathway [31]. There is also a report that Fasudil Dichloroacetate (FDCA) ameliorates hypoxia-induced pulmonary arterial smooth muscle cell (PASMC) dysfunction by inhibiting both CaMK and Rho-kinase signaling pathways, as well as maintaining mitochondrial homeostasis, thus alleviating SU5416 plus hypoxia (SuHx)-induced pulmonary arterial hypertension (PAH) [32]. Our study demonstrates NCC phosphorylation via CaMKII phosphorylation in murine distal convoluted cells (mDCT15 cells) under CoCl_2_-induced hypoxic conditions, contributing to the understanding of how hypoxia regulates NCC activity through the CaMKII pathway. Interestingly, however, a recent study reported that pharmacological inhibition of CaMKII using the compound KN93 leads to an increase in the active, phosphorylated form of NCC at the luminal membrane, as well as enhanced NCC activity in murine DCT15 cells [33]. This discrepancy suggests that the regulation of NCC by CaMKII may be context-dependent, with hypoxic conditions and simple pharmacological interventions affecting NCC in different ways via CaMKII-β.

The regulation of NCC by hypoxia and CaMKII has significant implications for renal physiology and the pathophysiology of hypertension. Enhanced NCC activity can lead to increased sodium reabsorption, contributing to fluid retention and elevated blood pressure. Understanding the molecular mechanisms underlying NCC regulation in hypoxic conditions can provide insights into the development of therapeutic strategies for conditions such as chronic kidney disease and hypertension. As an important molecule in NCC regulation, CaMKII may also be a new therapeutic target to affect NCC expression by regulating CaMKII activity, therefore improving kidney function.

However, there are some limitations to our study. First, even though CoCl_2_ is a common hypoxia mimetic, it does not fully replicate the complexity of in vivo hypoxia. Future studies should consider employing other models and in vivo experiments to verify our findings. Additionally, the precise molecular interactions between HIF-1α downstream targets, CaMKII, and NCC require further investigation.

In conclusion, our study demonstrates that CoCl_2_-induced hypoxia increases NCC expression and phosphorylation via a CaMKII-dependent pathway in mDCT15 cells. The inhibition of CaMKII significantly reduces NCC expression, highlighting the critical role of CaMKII in this regulatory mechanism. These findings contribute to our understanding of how hypoxia influences renal ion transport and provide a foundation for future research into therapeutic strategies for hypertension and related disorders.

## Data Availability

The original contributions presented in the study are included in the article, further inquiries can be directed to the corresponding author.

## Abbreviations

DCT	Distal convoluted tubule
NCC	Sodium-chloride co-transporter
CoCl_2_	Cobalt chloride
CaMKII	Calcium/calmodulin-dependent protein kinase II
mDCT15	mouse DCT15
PHD	prolyl hydroxylases
pVHL	von Hippel-Lindau tumor-suppressor protein
KN93	N-[2-[N-(4-chlorocinnamyl)-N-methylaminomethyl]phenyl]-N-(2-hydroxyethyl)-4-methoxybenzenesulfonamide
DMSO	Dimethyl sulfoxide
HIF-1α	Hypoxia-Inducible Factor-1α
p-NCC	phosphorylated NCC
lcn2 KO	lcn2 knockout
WT	Wild-type
CaM	Calmodulin
SAHA	Suberoylanilide hydroxamic acid
I/R	Ischemia-reperfusion
NCX	Na⁺-Ca^2^⁺ exchanger
FDCA	Fasudil Dichloroacetate
PAH	Pulmonary arterial hypertension
SuH	SU5416 plus hypoxia
PASMC	Pulmonary arterial smooth muscle cell
